# Epidemiological and Etiological Characteristics of Fever, Thrombocytopenia and Leukopenia Syndrome in Henan Province, China, 2011–2012

**DOI:** 10.1371/journal.pone.0091166

**Published:** 2014-03-14

**Authors:** Xueyong Huang, Yanhua Du, Xiaoning Hu, Hongxia Ma, Haifeng Wang, Aiguo You, Kai Kang, Haomin Chen, Li Zhang, Guohua Liu, Bianli Xu

**Affiliations:** 1 Henan Center for Disease Control and Prevention, Zhengzhou, China; 2 College of Public Health, Zhengzhou University, Zhengzhou, China; 3 Xinyang Center for Disease Control and Prevention, Xinyang, China; Institut Pasteur of Shanghai, Chinese Academy of Sciences, China

## Abstract

The Fever, Thrombocytopenia and Leukopenia Syndrome (FTLS) is caused by a bunyavirus known as the FTLS virus (FTLSV), which was recently discovered in China. We examined the epidemiological and etiological features of 637 laboratory-confirmed cases of FTLS with onset from January 2011 to December 2012 in Henan Province, China. The highest incidence of FTLS occurred between May and August: 76.5% of all laboratory-confirmed cases occurred during those four months. Of the laboratory-confirmed cases, 60.9% were in the 46–69 years old age groups; 96.1% (612/637) occurred in farmers; 98.1% (625/637) were reported from Xinyang Prefecture. During the same time period, 2047 cases were reported in China. The nucleotide and amino acid sequences of FTLSV strains identified during 2011–2012 in Henan Province were ≥96% identical. This findings provides insight for developing public-health interventions for the control and prevention of FTLS in epidemic area.

## Introduction

The Fever, Thrombocytopenia and Leukopenia Syndrome virus (FTLSV), also known as the Severe Fever with Thrombocytopenia Syndrome virus (SFTSV) and Huaiyangshan virus, belongs to the *phlebovirus genus*, the family of *Bunyaviridae* [1]–[3]. Likes other bunyaviruses [4]–[6], the L segment encodes RNA-dependent RNA polymerase; the M segment has an open reading frame (ORF) coding for a GnGc precursor in the order Gn-Gc; whereas the S segment uses ambisense coding to express two proteins: one is a nucleocapsid (N) protein encoded by the 5′ half of viral complementary sense S RNA, and the other is a nonstructural (NS) protein encoded by viral sense S RNA [7]. The virus has been found to cause the Fever, Thrombocytopenia and Leukopenia Syndrome (FTLS), also known as the Severe Fever with Thrombocytopenia Syndrome (SFTS) and Huaiyangshan Hemorrhagic Fever in China since 2007 [1]–[3]. The disease has been reported in 11 Chinese provinces (Henan, Hubei, Shandong, Anhui, Jiangshu, Liaoning, Yunnan, Guangxi, Jiangxi, Shanxi and Zhejiang Provinces) and Shanghai City [1]–[3], [8], [9]. 2047 cases of FTLS were reported during 2011–2012 in China. Henan Province has the highest case count, and accounts for 48.2% of all reported cases in China [10]. Recently, this virus has also been found to cause severe disease and death in other countries such as Japan and Korea [11]. Major clinical presentations of FTLS included fever, thrombocytopenia, leukocytopenia, gastrointestinal symptoms, neurological symptoms, bleeding tendency, as well as less specific clinical manifestations. It is difficult to differentiate FTLS from other infectious diseases, especially hemorrhagic fever with renal syndrome and human granulocytic anaplasmosis [1]. This disease has a case-fatality rate ranging from 2.5% to 30% in different areas of endemicity [12]. No vaccine or antiviral drugs are currently available [13]. FTLS has been a Category B reportable infectious disease in mainland China since September 29, 2010.

Using surveillance data from January 1, 2011 to December 31, 2012 in Henan Province, we studied the epidemiological and etiological characteristics of FTLS, to inform the development of effective prevention and control measures.

## Materials and Methods

### Case Definitions and Specimen Collection

All clinically diagnosed FTLS cases in Henan Province are reported to the Henan Center for Disease Control and Prevention by medical practitioners, who make the clinical diagnosis based on the guidelines by the National Health and Family Planning Commission [1], [14], as follows: (1) Epidemiological characteristics, including history of tick bites, working in the mountainous areas or the forest, or direct contact with the blood of a confirmed case-person during the two weeks prior to symptom onset; (2) Clinical presentations, including at least one of the following: Fever, headache, muscle aches, nausea, vomiting, diarrhea, skin bruises, bleeding, multiple organ damage, disseminated intravascular coagulation (DIC); (3) Laboratory findings, including decrease of leukocyte count and thrombocytopenia; and (4) Ruling out other diseases, such as human granulocytic anaplasmosis, hemorrhagic fever with renal syndrome, dengue fever, thrombocytopenic purpura, septicaemia. A patient who met all of the above criteria was a clinically diagnosed FTLS case-patient. We collected and tested blood specimens from clinically diagnosed FTLS case-patients who were hospitalized. Both acute and convalescent phase sera were collected by hospital staff and transported to the laboratories using cold chain. A laboratory-confirmed case-patient met at least one of the following criteria: Isolation of SFTSV from the patient’s serum; detection of FTLSV RNA in the patient’s serum during the acute phase of the illness; detection of anti-FTLSV IgM antibody by enzyme-linked immunosorbent assay (ELISA); or seroconversion or a four-fold elevation in serum IgG antibodies against FTLSV by indirect immunofluorescence assay (IFA). We analyzed the data for laboratory-confirmed FTLS case-patients to assess their epidemiological and etiological characteristics.

We used a structured questionnaire to collect the case-patients’ sociodemographic and clinical data. The data were verified by checking against medical records.

### Real-time RT-PCR Detection

Total RNA was extracted from acute serum samples using a QIAamp viral RNA mini Kit (Qiagen, Germany), following the manufacturer’s instructions. The real-time RT-PCR assay was performed using primers (S3-137F, 5′-ATGGATGCAAGGTCCAGAAT-3′, S3-268R, 5′-CTTCCTCAGAGCTGCTTGCT-3′), probe (C437-PROBE2, 5′-TGCCGCTGAGCAATTTCTCTC TCCA-3′) [15] and the QIAGEN QuantiTectTM Probe RT-PCR Kit (Qiagen, Germany). The conditions for real-time RT-PCR reaction were as follows: 50°C for 30 min, 95°C for 15 min, 45 cycles of 95°C for 15 s,60°C for 45 s. Data were analyzed using the software supplied by the manufacturer.

### ELISA Assay

Anti-FTLSV IgM antibodies were detected in acute-phase sera of case-patients using an ELISA kit (Xinlianxin Biomedical Technology CO., LTD, Wuxi, Jiangsu, China) according to the manufacturer’s protocol. For this ELISA kit, we used 50 µl of positive control, negative control, and sera diluted to 1∶40. The test results were determined to be valid if the criteria for the positive control and the negative controls were fulfilled. The absorbance values of the positive control must be more than 1.5 at 450 nm after blanking; the absorbance values of the negative control must be less than 0.1 at 450 nm after blanking. If the absorbance values of the controls did not meet the quality control range specifications, the test was determined to be invalid and must be repeated. Calculation of the cut-off value was the mean absorbance value for negative controls times 2.1; if the mean absorbance value for negative controls was lower than 0.05, then the value 0.05 was used. The positive/negative determination of samples was performed using the cut-off value.

### Indirect IFA

FTLSV-specific IgG antibodies were detected in the patients’ sera during the acute and convalescent phases by indirect IFA, as previously described [16]. Vero E6 cells-infected FTLSV showing cytopathic effect (CPE) were harvested and centrifuged at 1,000×g for 10 minutes. The centrifugal precipitate was re-suspended in 1×PBS, spotted onto 12-well glass slides, and fixed with acetone for 10 minutes. Two-fold serial dilutions (1∶10 to 1∶1280) of the sera were prepared in PBS buffer. A volume of 20 µl of each serum sample was added to the cell-spotted wells and incubated for 45 minutes at 37°C. After washing for 10 minutes in PBS, 20 µl of FITC-conjugated goat anti-human IgG (Sihuan Sci-Technics Company, Beijing, China) diluted 1∶40 in buffer containing Evans Blue was added to each well and incubated for 30 minutes at 37°C. After washing, slides were mounted in glycerin and examined by immunofluorescence microscopy.

### Virus Isolation and Sequencing

All acute phase serum samples were used to inoculate Vero E6 cells. 100 µl of serum was inoculated onto Vero E6 cell monolayers in 25 cm^2^ flasks and incubated for 14 days at 37°C/5% CO_2_ in MEM/2% fetal calf serum with a media change after 7 days. All cultures were monitored daily for CPE. Each sample underwent at least three cell culture passages in Vero E6 cells before being considered negative [17]. Both virus-infected cells and negative control cells were examined for FTLSV by real-time RT-PCR at each passage. The whole genome sequences of FTLSV were amplified using primers described in previous studies by RT-PCR [3]. The RT-PCR products were sent to Sangon Biotech Co., Ltd (Shanghai, China) for DNA sequencing using an automated ABI 3730 DNA sequencer.

### Phylogenetic Analysis

The genomes of FTLSV isolates were compiled using the SeqMan program in the LaserGene software package (DNASTAR). The percentage similarities of nucleotide identity or amino acid identity were calculated using the Clustalx software. Molecular phylogenetic analysis was conducted by using the maximum likelihood (ML) method based on the Kimura 2-parameter model in the MEGA 5 software [18]. The tree with the highest log likelihood was shown. The percentage of trees in which the associated taxa clustered together was shown next to the branches. Initial tree(s) for the heuristic search were obtained automatically as follows: When the number of common sites was <100 or <1/4 of the total number of sites, the Maximum Parsimony method was used; otherwise the BIONJ method with MCL distance matrix was used. The tree was drawn to scale, with branch lengths measured in the number of substitutions per site. All available nucleotide sequences of genome segments of SFTSV isolates from GenBank were analyzed, together with our newly generated genome of SFTSV isolates in this study, and the phylogenetic trees were constructed in order to understand the evolutionary characterization of SFTSV.

### Statistical Analysis

All epidemiologic and laboratory data were double-entered with the EpiData 3.1 software. All statistical analyses were performed using SAS v9.13 (SAS Institute Inc., Cary, NC). An association with p<0.05 was considered to be statistically significant.

### Ethics Consideration

This research was approved by the Institutional Review Board at the Center for Disease Control and Prevention of Henan Province. All participants gave written informed consent for use of their samples for research purposes.

## Results

### Laboratory Findings

During January 2011 to December 2012, a total of 987 clinically diagnosed FTLS case-patients were reported; 812 (82.3%) of those patients gave acute phase sera and 529 (53.6%) gave convalescent phase sera. Of the 812 acute phase sera, 589 were positive for FTLSV RNA (using real-time RT-PCR) and/or anti-FTLSV IgM antibodies (using ELISA); these included 316 positive sera using both methods, 230 using FTLSV RNA only, and 43 using anti-FTLSV IgM antibodies only. An additional 48 paired sera had positive anti-FTLSV IgG antibody by the IFA test (including 29 seroconversions and 19 with ≥4-fold increase). In total, there were 637 ( = 589+48) laboratory-confirmed case-patients in this study.

### Epidemiology Characteristics

#### Place

**Figure 1:**
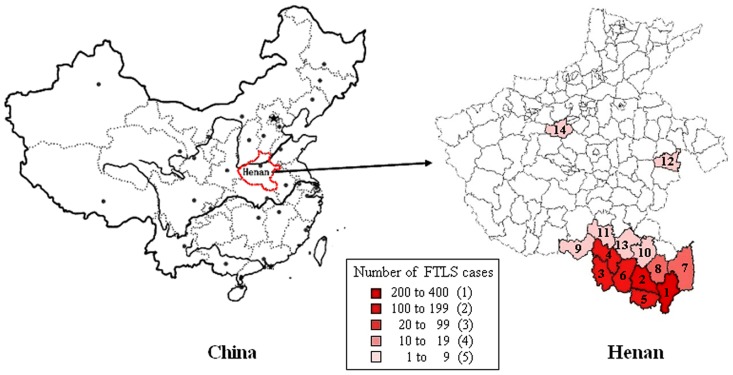
Map of Henan Province, showing laboratory-confirmed FTLS cases in 2011–2012. 1 Shangcheng, 2 Guangshan, 3 Sihe), 4 Pingqiao, 5 Xinxian, 6 Luoshan, 7 Gushi, 8 Huangchuan, 9 Tongbai, 10 Xixian, 11 Queshan, 12 Luyi, 13 Zhengyang, 14 Dengfeng.

The annual incidence rate based on the laboratory-confirmed cases was 0.35 per 100,000 population in Henan Province. The vast majority (98.1%, 625/637) of the laboratory-confirmed cases were reported in Xinyang Prefecture in southern Henan Province (Figure 1).

#### Time

**Figure 2:**
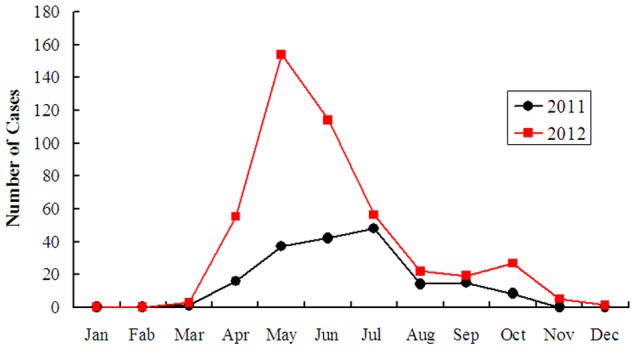
Monthly distribution of reported FTLS cases: Henan Province, China, January 2011 to December 2012.

FTLS showed strong seasonality. In both 2011 and 2012, cases started to increase in March, and peaked between May and August; cases during those four months accounted for 76.5% (487/637) of all laboratory-confirmed cases. The monthly case count peaked in July during 2011 and May during 2012. Cases started to decline in October in both years (Figure 2).

#### Person

Of the 637 laboratory-confirmed case-patients, 40.2% (256/637) were males and 59.8% (381/637) were females. The median age of the case-patients was 60 (range, 10–90) years; 60.9% (388/637) of the patients were 46–69 years in age. The sex distribution differed significantly among the age groups (χ^2^ = 147.8553, P<0.0001). The vast majority of the case-patients (96.1%, 612/637) were farmers, including agricultural and forest workers living in rural areas.

#### Deaths

During 2011 and 2012, 18 deaths due to FTLS were reported in Henan Province; the case fatality rate was 2.8% (18/637). The median age of deceased case-patients was 66.5 (range, 42–77) years. The mortality rate increased with age (Cochran-Armitage trend test, Z = 2.3622, P = 0.0182).

### Molecular Characterization of FTLSV Strains

To characterize the FTLSV strains circulating in Henan Province and investigate their genetic origin, we analyzed the viruses isolated from acute serum samples tested positive for FTLSV RNA. Of the 133 FTLSV strains isolated and confirmed by real time RT-PCR, complete genomes were determined for 10 strains isolated during 2011 and 19 strains isolated during 2012. The GenBank accession numbers of SFTSV obtained in this study were listed in Table 1.

**Table 1:** The GenBank accession numbers of SFTSV obtained in this study.

Name of FTLSVstrain	GenBank accessionNO. of L segment	GenBank accessionNO. of M segment	GenBank accessionNO. of S segment	Isolationsource	geographic origin
FLSV2011YPQ11	KF711886	KF711926	KF711897	Human serum	pingqiao
FLSV2011YPQ12	KF711880	KF711930	KF711915	Human serum	pingqiao
FLSV2011YPQ17	KF711887	KF711923	KF711896	Human serum	pingqiao
FLSV2011YGS22	KF711888	KF711927	KF711894	Human serum	guangshan
FLSV2011YGS45	KF711870	KF711925	KF711893	Human serum	guangshan
FLSV2011YGS5	KF711884	KF711945	KF711899	Human serum	guangshan
FLSV2011YGS52	KF711861	KF711922	KF711895	Human serum	guangshan
FLSV2011YGS60	KF711863	KF711947	KF711900	Human serum	guangshan
FLSV2011YGS7	KF711885	KF711928	KF711898	Human serum	guangshan
FLSV2011YXX9	KF711889	KF711924	KF711909	Human serum	xinxian
FLSV2012YGS10	KF711872	KF711937	KF711904	Human serum	guangshan
FLSV2012YGS4	KF711875	KF711933	KF711902	Human serum	guangshan
FLSV2012YSC6	KF711879	KF711931	KF711911	Human serum	shangcheng
FLSV2012YSH10	KF711868	KF711940	KF711912	Human serum	pingqiao
FLSV2012YSH104	KF711867	KF711943	KF711914	Human serum	shangcheng
FLSV2012YSH105	KF711869	KF711944	KF711916	Human serum	shihe
FLSV2012YSH107	KF711864	KF711942	KF711910	Human serum	shihe
FLSV2012YSH14	KF711876	KF711938	KF711908	Human serum	shangcheng
FLSV2012YSH16	KF711874	KF711934	KF711903	Human serum	shangcheng
FLSV2012YSH27	KF711878	KF711932	KF711905	Human serum	shihe
FLSV2012YSH37	KF711871	KF711936	KF711918	Human serum	guangshan
FLSV2012YSH6	KF711882	KF711921	KF711892	Human serum	xinxian
FLSV2012YSH86	KF711877	KF711939	KF711907	Human serum	gushi
FLSV2012YSH89	KF711873	KF711935	KF711906	Human serum	shangcheng
FLSV2012YSH9	KF711881	KF711919	KF711890	Human serum	xinxian
FLSV2012YSH91	KF711883	KF711920	KF711891	Human serum	shihe
FLSV2012YSH92	KF711865	KF711941	KF711917	Human serum	shihe
FLSV2012YSH93	KF711866	KF711946	KF711913	Human serum	shihe
FLSV2012YXX1	KF711862	KF711929	KF711901	Human serum	xinxian

The nucleotide sequences of FTLSV isolates in this study were closely related to each other, with 95.8% to 99.9% nucleotide identity for the complete L segments, 98.0% to 100% nucleotide identity for the complete M segments, and 95.1% to 99.9% nucleotide identity for the partial S segments. The identity of amino acid sequences was 98.8% to 100% in RNA-dependent RNA polymerase, 96.6% to 100% in GnGc precursor, and 98.3% to 100% in N protein, respectively.

**Figure 3:**
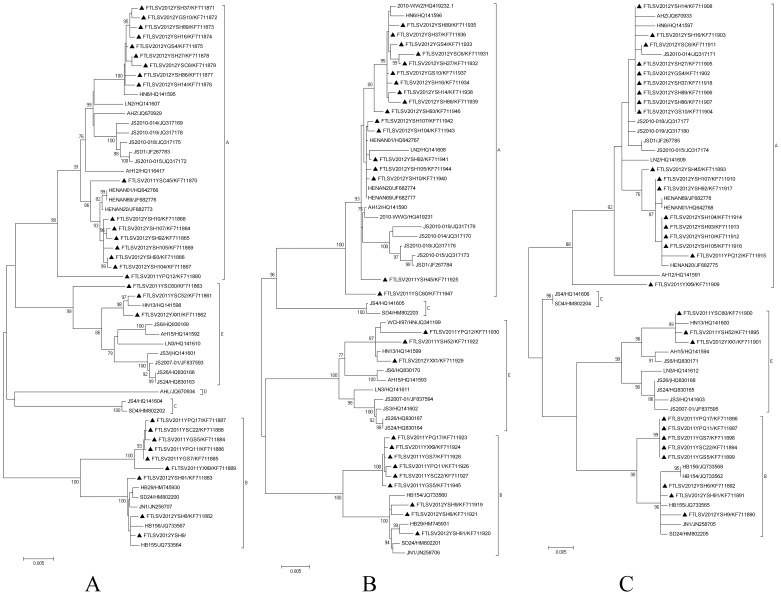
Phylogenetic analysis of FTLSV strains isolated from Henan Province during 2011 and 2012, compared with other bunyaviruses. The phylogenetic tree was constructed by using the maximum likelihood method with the MEGA5 software. The reliability values indicated at the branch nodes were determined using 1,000 bootstrap replications. Isolated FTLSV strains in 2011 and 2012 from Henan Province were labeled by black solid triangles. Phylogenetic relationship of FTLSV with other bunyaviruses, based on the complete L, M, S segment sequences, are shown in panel A, B, C, respectively.

Using the ML method in MEGA software, phylogenetic trees were constructed with the sequences of 29 isolates in this study and 27 reference FTLSV strains from GenBank. Phylogenetic analysis of the L segment sequences revealed that the FTLSV strains were classified into four large groups in a previous study: A, B, C and E [19]. Our new isolates belonged to groups A, B, and E. Most of the 2011 isolates belonged in Group A, whereas most of the 2012 isolates belonged in Group B; a small number of strains belonged to Group E. The trees based on the S or M segment sequences had similar topologies as those based on the L segment sequences (Figure 3).

## Discussion

From January 2011 to December 2012, 987 clinically diagnosed FTLS cases were reported in Henan Province. Although cases were detected each month, nearly three-quarters occurred during May-August, suggesting that surveillance and prevention efforts should be strengthened during summer months each year. The incidence rate was highest among persons aged 46–69 years; most of reported case-patients were farmers living in wooded and hilly areas or working in the fields. Some FTLS case-patients reported a history of tick bite before illness. Previously, FTLSV RNA had been detected in some ticks, and FTLSV stains were isolated from ticks collected from dogs and goats in the epidemic region [20]. Tick densities in the epidemic areas in Henan Province are high during May-August, coinciding with the peak of the cases [21], indicating that FTLSV may be mostly tick-borne. Therefore, prevention of tick bites should be promoted as important prevention and control measures to reduce FTLS in the epidemic area.

The vast majority of FTLS cases were reported in Xinyang Prefecture in southern Henan Province (114°01′–114°06′ E, 31°46′–31°52′ N). Of the 6,108,683 population in Xinyang Prefecture, 85% work in agriculture. The county consists of southwestern mountains, a central hilly district, and northern plains and depressions. It is in a subtropical to warm-temperate transitional zone, with a continental monsoon humid climate and 4 distinct seasons (annual average temperature: 15.2°C; average rainfall: 1,150 mm). The major sources of income for the farmers in this county included various crops, fruit trees, tea, and livestock breeding. These features create ideal conditions for tick-borne diseases such as FTLS to spread.

The high (>96%) identity of the virus isolates from the laboratory-confirmed FTLS cases during the study period indicated a lack of variability in the FTLSV strains circulating in Henan Province. Of these diagnostic methods, virus isolation is the most time-consuming, and requires expensive instruments or special techniques. Real-time RT-PCR and IFA can be completed in 2–4 h [22]–[24], but this technique requires elaborate methods for the detection of amplified products or sophisticated instruments. ELISA assays could offer a simple, convenient and efficient method of measuring antibodies in large-scale epidemiology studies in a wide range of settings, without requiring specialized and expensive measuring equipments [25], [26]. ELISA assays may also be applied in early diagnosis for FTLS patient.

The epidemiology, pathogenesis and mode of transmission of FTLS are still unclear because this disease was only recently discovered. FTLS is a serious public health problem in Henan Province, causing many severe diseases and deaths annually. Our study describes the characteristics of FTLS cases and makes recommendations for prevention measures, which can help to inform the prevention and treatment of FTLS in the endemic area.

## References

[1] Xu BL, Liu LC, Huang XY, Ma H, Zhang Y, et al (2011) Metagenomic analysis of Fever, Thrombocytopenia and Leukopenia Syndrome (FTLS) in Henan Province, China: Discovery of a new Bunyavirus. Plos Pathogens 11: e1002369. 10.1371/journal.ppat.1002369 PMC3219706 22114553

[2] Zhang YZ, Zhou DZ, Xiong YW, Chen XP, He YW, et al (2011) Hemorrhagic fever caused by a novel tick-borne Bunyavirus in Huaiyangshan, China. Chin J Epidemiol 32: 209–220. 21457654

[3] Yu XJ, Liang MF, Zhang SY, Liu Y, Li JD, et al (2011) Fever with thrombocytopenia associated with a novel Bunyavirus in China. N Engl J Med 364: 1523–1532. 21410387 10.1056/NEJMoa1010095 PMC3113718

[4] Amman BR, Manangan AP, Flietstra TD, Calisher CH, Carroll DS, et al (2013) Association between movement and Sin Nombre virus (Bunyaviridae: Hantavirus) infection in North American deermice (Peromyscus maniculatus) in Colorado. J Wildl Dis 49: 132–142. 23307379 10.7589/2012-02-041

[5] Lihoradova OA, Indran SV, Kalveram B, Lokugamage N, Head JA, et al (2013) Characterization of Rift Valley fever virus MP-12 strain encoding NSs of Punta Toro virus or sandfly fever Sicilian virus. PLoS Negl Trop Dis 7: e2181. 23638202 10.1371/journal.pntd.0002181 PMC3630143

[6] Xiao H, Tian HY, Cazelles B, Li XJ, Tong SL, et al (2013) Atmospheric moisture variability and transmission of hemorrhagic fever with renal syndrome in changsha city, mainland china, 1991–2010. PLoS Negl Trop Dis 7: e2260. 23755316 10.1371/journal.pntd.0002260 PMC3674989

[7] Zhang YZ, Zhou DJ, Qin XC, Tian JH, Xiong Y, et al (2012) The ecology, genetic diversity, and phylogeny of Huaiyangshan virus in China. J Virol 86: 2864–2868. 22190717 10.1128/JVI.06192-11 PMC3302241

[8] Tommy TY, Liu W, Thomas AB, Cui N, Zhuang L, et al (2013) Evolutionary and molecular analysis of the emergent severe fever with thrombocytopenia syndrome virus. Epidemics 5: 1–10. 23438426 10.1016/j.epidem.2012.09.002 PMC4330987

[9] Pan H, Hu JY, Liu SL, Shen H, Zhu YY, et al (2013) A reported death case of a novel bunyavirus in Shanghai, China. Virology Journal 10: 1–5. 23758684 10.1186/1743-422X-10-187 PMC3689053

[10] Ding F, Zhang WY, Wang LY, Hu WB, Soares Magalhaes RJ, et al (2013) Epidemiologic features of severe fever with thrombocytopenia syndrome in China, 2011–2012. Clinical Infectious Diseases 56: 1682–1683. 23429379 10.1093/cid/cit100

[11] Chang MS, Woo JH (2013) Severe fever with thrombocytopenia syndrome: tick-mediated viral disease. J Korean Med Sci 28: 795–796. 23772137 10.3346/jkms.2013.28.6.795 PMC3677989

[12] Liu W, Lu QB, Cui N, Li H, Wang LY, et al (2013) Case-fatality ratio and effectiveness of ribavirin therapy among hospitalized patients in china who had severe fever with thrombocytopenia syndrome. Clin Infect Dis 57: 1292–1299. 23965284 10.1093/cid/cit530

[13] Yu L, Zhang L, Sun LN, Lu J, Wu W, et al (2011) Critical epitopes in the nucleocapsid protein of SFTS virus recognized by a panel of SFTS patients derived human monoclonal antibodies. PLoS ONE 7: e38291. 10.1371/journal.pone.0038291 PMC3373585 22719874

[14] (2010) National guideline for prevention and control of severe fever with thrombocytopenia syndrome. Beijing: National Health and Family Planning Commission of People’s Republic of China.

[15] Huang XY, Liu LC, Du YH, Ma H, Mu YJ, et al (2013) Detection of a novel bunyavirus associated with fever, thrombocytopenia and leukopenia syndrome in Henan Province, China, using real-time reverse transcription PCR. J Med Microbiol 62: 1060–1064. 23618800 10.1099/jmm.0.049577-0

[16] Huang XY, Du YH, Li XL, Ma H, Man RQ, et al (2012) Establishment of indirect immunofluorescence assay (IFA) for detection of IgG antibody against new bunyavirus. Chin J Prev Med 46: 165–168. 22490201

[17] Du YH, Huang XY, Deng WB, Ma HX, Ma H, et al (2012) Culture, isolation and identification of new bunyavirus in African green monkey kidney (Vero) cells. Chin J Prev Med 46: 169–172. 22490202

[18] Tamura K, Dudley J, Nei M, Kumar S (2007) MEGA4: molecular evolutionary genetics analysis (MEGA) software version 4.0. Mol Biol Evol pp: 1596–1599. 10.1093/molbev/msm092 17488738

[19] Zhang YZ, He YW, Dai YA, Xiong Y, Zheng H, et al (2012) Hemorrhagic fever caused by a novel Bunyavirus in China: pathogenesis and correlates of fatal outcome. Clin Infect Dis 54: 527–533. 22144540 10.1093/cid/cir804

[20] Jiang XL, Wang XJ, Li JD, Ding SJ, Zhang QF, et al (2012) Isolation, identification and characterization of SFTS bunyavirus from ticks collected on the surface of domestic animals. Chin J Virol 28: 252–257. 22764528

[21] Liu Y, Huang XY, Du YH, Wang HF, Xu BL (2012) Survey on ticks and detection of new bunyavirus in some vect in the endemic areas of fever, thrombocytopenia and leukopenia syndrome(FTLS) in Henan province. Chin J Prev Med 46: 500–504. 22943894

[22] Neeraja M, Lakshmi V, Dash PK, Parida MM, Rao PV (2013) The clinical, serological and molecular diagnosis of emerging dengue infection at a tertiary care institute in southern India. J Clin Diagn Res 7: 457–461. 23634396 10.7860/JCDR/2013/4786.2798 PMC3616556

[23] Lam WY, Leung TF, Lee N, Cheung JL, Yeung AC, et al (2010) Development and comparison of molecular assays for the rapid detection of the pandemic influenza A (H1N1) 2009 virus. J Med Virol 82: 675–683. 20166184 10.1002/jmv.21725

[24] Reddy V, Ravi V, Desai A, Parida M, Powers AM, et al (2012) Utility of IgM ELISA, TaqMan real-time PCR, reverse transcription PCR, and RT-LAMP assay for the diagnosis of Chikungunya fever. J Med Virol 84: 1771–1778. 22997080 10.1002/jmv.23406

[25] Ojogun N, Kahlon A, Ragland SA, Troese MJ, Mastronunzio JE, et al (2012) Anaplasma phagocytophilum outer membrane protein A interacts with sialylated glycoproteins to promote infection of mammalian host cells. Infect Immun 80: 3748–3760. 22907813 10.1128/IAI.00654-12 PMC3486060

[26] Martin RM, Patel R, Zinovik A, Kramer MS, Oken E, et al (2012) Filter paper blood spot enzyme linked immunoassay for insulin and application in the evaluation of determinants of child insulin resistance. PLoS One 7: e46752. 23056434 10.1371/journal.pone.0046752 PMC3466324

